# Challenges, uncertainties and perceived benefits of providing weekend allied health services—a managers’ perspective

**DOI:** 10.1186/s12913-017-2035-4

**Published:** 2017-02-06

**Authors:** Deb Mitchell, Lisa O’Brien, Anne Bardoel, Terry Haines

**Affiliations:** 1 Allied Health Workforce, Innovation, Strategy, Education and Research Unit, Monash Health, Dandenong, Victoria Australia; 2 Department of Physiotherapy, School of Primary Healthcare, Monash University, Frankston, Victoria Australia; 3 Department of Occupational Therapy, School of Primary Healthcare, Monash University, Frankston, Victoria Australia; 4 Department of Management, Faculty of Business and Economics, Monash University, Caulfied East, Victoria Australia; 5 Allied Health Research Unit, Monash Health, Cheltenham, Victoria Australia

## Abstract

**Background:**

Health services that operate 7 days per week are under pressure to show the increased cost of providing weekend services can be measured in improved patient outcomes. The evidence for weekend allied health services in acute medical and surgical wards is weak and there is wide variation between the services offered at different hospitals.

**Methods:**

This qualitative study was undertaken during a multi-site stepped wedge randomised controlled trial involving twelve acute medical and surgical wards from two Australian hospitals, in which weekend allied health services were removed before being reinstated with a stakeholder driven model. In-depth interviews were conducted with twenty-two staff responsible for managing weekend services at the involved hospitals. Participants were asked about their perceptions of the advantages and disadvantages of providing a weekend allied health service.

**Results:**

Managers perceive the services improve patient flow and quality of care and reduce adverse incidents, such as falls and intensive care admissions. They also highlighted the challenges involved in planning, staffing and managing these services and the uncertainties about how to provide it most effectively.

**Conclusions:**

Rising healthcare costs provide opportunity for public and professional debate about the most effective way of providing weekend allied health care services, particularly when health services provide limited other weekend services. Some managers perceived weekend allied health services to improve patient quality of care, but without studies which show these services on acute medical and surgical wards clearly change patient outcomes or provide health economic gains, these resources may need to be redirected. The resources may be better spent in areas with clear evidence to show the addition of weekend allied health services improves patient outcomes, such as on acute assess units and rehabilitation wards.

## Background

Hospitals face competing demands to provide high quality care, 7 days per week, while also minimising the costs of service delivery [1]. Providing the same amount of care on weekends as during the week is problematic as it is a time when some staff are reluctant to work and industrial agreements in some countries dictate that rates of pay are higher for work done on weekends [2–4]. Minimising cost by reducing services or employing junior staff on the weekend may result in poorer outcomes for patients [5–7]. A “weekend effect” [8] has been reported, where patients admitted on the weekend have worse health outcomes than those admitted during the week, with higher rates of adverse events such as falls [1, 9–13] and death [14]. This indicates that the provision of services similar to weekday services may be justified on weekends.

Allied health professionals and assistants are a group that may provide weekend services. Disciplines involved may include dietitians, occupational therapists, physiotherapists, social workers and speech pathologists, complemented by allied health assistants. Potential roles include discharge planning, chest physiotherapy, and the assessment of diet, mobility, and swallowing. There appears to be wide variation between the amount and type of weekend allied health services provided between hospitals, with much of the research examining the provision of physiotherapy services. Shaw et al. found that 61% of Australian hospitals provide physiotherapy services on Saturdays and 45% on Sundays [15]. In Canada, 87% of hospitals provide some type of weekend physiotherapy services [16]. Whilst there is some support for the effectiveness of physiotherapy and occupational therapy on weekends in rehabilitation settings [17–21], there is minimal evidence from acute hospital settings.

Providing a weekend allied health service in acute hospitals is potentially problematic. There are a variety of models that can be pursued, different disciplines employed, and different tasks they can perform. The individual needs of patients on different types of wards will differ, indicating a “one-size-fits-all” approach is inappropriate. This creates great difficulty for managers in the design and provision of these services. As yet there has been no in-depth investigation of the challenges and uncertainties faced, nor the benefits perceived by allied health managers in designing and providing weekend allied health services on acute medical and surgical wards. This paper aims to address this gap so weekend allied health services can be better designed, provided, managed, and evaluated in future. This paper was guided by the research question: “What are the perceptions of allied health managers regarding the day to day management and training of staff that provide weekend allied health services on acute medical and surgical wards?”

## Method

### Design

The overarching research design was descriptive qualitative [22] in which the experiences of the research group informed the exploration of the perceptions of the managers of staff who provide weekend allied health services. These managers are the gatekeepers who decide how these services are resourced and staffed. This qualitative study involved semi-structured interviews conducted in either one-to-one or small group (up to six participants) interview formats. It was conducted during the first phase of a multi-site stepped wedge randomised controlled trial that commenced in February 2014 in which existing weekend allied health services were removed as a part of the trial [23]. Ethical approval for the removal of these services was obtained from each hospital’s ethics committee.

### Participants and settings

Participants in this research were allied health managers (*n* = 11) or senior clinicians (*n* = 11) who oversaw weekend allied health services affected by the broader trial (Table 1). These managers at the trial sites were targeted as we felt that having their weekend allied health service removed as a part of the broader trial would have primed them to have a rich and expansive discussion of this topic. We interviewed the managers of existing weekend allied health services at each site and allied health managers whose services may potentially have been implemented in the second half of the larger study. Senior clinical staff were interviewed as they are routinely delegated the day to day management and training of weekend allied health staff.

**Table 1:** Study participants

	Allied health managers	Clinicians with responsibility for managing weekend services
Hospital A	*n* = 7	*n* = 5
Age 30–40	1	3
Aged 40–50	3	1
Aged 50 +	3	1
Hospital B	*n* = 4	*n* = 6
Aged 20–30	0	3
Age 30–40	1	3
Aged 40–50	1	
Aged 50 +	2	

The hospitals involved (denoted by Hospital A and Hospital B) were acute tertiary hospitals in Melbourne, Victoria, Australia. Weekend allied health services on the acute medical and surgical wards at these hospitals were different. Hospital A provided dietetics, occupational therapy, physiotherapy, speech pathology, social work and allied health assistant services to its acute medical and surgical wards on the weekends, whereas Hospital B provided occupational therapy and physiotherapy only. The hours of weekend service at Hospital A was approximately twenty percent of the weekday service and at Hospital B, approximately 10%.

### Procedure

We developed a semi-structured interview schedule (see Appendix) with questions aimed at exploring the service each discipline provided to the acute medical and surgical wards, the advantages (including perceived benefits) and disadvantages, including the challenges faced, when managing weekend services. The questions were refined after initial interviews to further probe apparent patterns or themes. Participants were asked to focus their feedback on weekend allied health services in acute medical and surgical wards in accordance with the scope of the larger study, but all disciplines also provided services to other parts of the hospital, such as the intensive care unit and the emergency department.

The research design, including the planned key informant interviews, was explained to allied health managers at operational meetings at each hospital. Managers and, where applicable, senior clinicians to whom the day to day management of the weekend allied health was delegated, were identified for interview by the research site liaison at each hospital and then approached by email. All managers agreed to participate and an interview time was arranged. All interviews except for two were conducted in person, with the remainder being conducted by telephone. Participants gave informed, written consent to participate in the study and for the results of the study to be published, prior to the interview, and were informed that they could withdraw this consent at any time. Most interviews were conducted individually, however in some instances the managers and senior clinicians preferred to be interviewed in pairs to allow for timely collection of their feedback. At Hospital B, a group of clinicians were responsible for training the weekend staff and they were interviewed as a group. The breakdown of interviews versus focus groups can be found in Table 2. All interviews were facilitated by DM, digitally recorded, and transcribed verbatim. Ethical approval for the study was obtained from each hospital’s ethics commitee.

**Table 2:** Interveiwer : Participant ratio

Interviewer: Participant ratio	Number of interviews
1:1	8
1:2	4
1:6	1

### Analysis

Recordings and transcripts of interviews and focus groups were loaded into nVivo software version 10 (QSR International). Thematic analysis methods were used to code and group the data [24, 25]. After each interview, memos were recorded and new questions added to further explore emerging ideas during subsequent data collection. After familiarisation with the data, DM identified initial codes and a developed a coding matrix to which the data was assigned These codes were then examined for overarching categories, which focussed on the managers perceptions of the advantages and disadvantages of providing a weekend allied health service. In moving between the data and categories, the data began to make sense in the light of researchers’ own experiences of weekend allied health service provision and the advantages and disadvantages could then be grouped into themes. We defined themes as a range of views which reflect a participant’s preoccupation with an issue. Explanatory accounts were then developed to reflect the meaning of the original data, explaining the emerging themes and seeking wider application of these themes [26]. Interpretations were discussed with other researchers (LOB, AB and TH) for clarity and trustworthiness and disagreements were resolved by discussion.

Themes were then member checked with representatives of the participant groups for accuracy and authenticity. At the request of one of the participants, one quotation was moved into another theme and background information was added to put one of the quotes in context. We also removed reference to which hospital participants were from, to protect their anonymity.

### Background, training and preconceptions of investigators

All investigators except one, have backgrounds in allied health. None were currently working on weekends. DM and TH are employed by one of the participating hospitals and are colleagues of the staff being interviewed at that site. All except one, were also investigators on the larger study investigating the effectiveness and cost effectiveness of weekend allied health services in acute medical and surgical wards. The lead investigator of this qualitative study was a PhD candidate with experience in running focus groups and key informant interviews and collating information from participants as part of project management.

Prior to the interviews, investigators anticipated that managers of weekend allied health services may magnify their value and diminish the challenges faced in managing these services in order to preserve the funding allocated to these services. To reduce the potential of the managers overstating the advantages of the service, equal numbers of questions were asked about the perceived advantages and disadvantages of providing the service and an investigator with a business management background was asked to check the manuscript for bias. None of the investigators manage or work in weekend allied health services, reducing the potential for conflict of interest.

## Results

Eleven allied health managers and eleven senior allied health clinicians participated in this study in twelve interviews and one focus group (Table 1). All allied health managers and senior clinicans had the same professional background as the staff they managed, except the allied health assistants at Hospital A were managed through physiotherapy. Interviews were held with managers from the current professions providing weekend allied health services at each site and with professions that could potentially provide weekend services in phase two of the larger study. At Hospital A we interviewed the dietetics, occupational therapy, physiotherapy, podiatry, speech pathology, and social work managers. At Hospital B we interviewed the dietetics, occupational therapy, physiotherapy and speech pathology managers. To protect the identities of participants, all will henceforth be referred to as managers. Data analyses revealed several challenges and uncertainties faced by allied health managers when planning weekend allied health services, primarily concerning the cost.

Three major themes were identified: uncertainty about whether the overall benefits justify the costs; uncertainty about how best to structure the service; and the challenges involved in running a service outside of business hours.

### Key uncertainty 1) Do the overall benefits justify the costs?

The primary uncertainty faced by the managers was whether the actual cost of providing a service was justified by the benefits attained from having it. Managers identified a number of potential benefits including reduced patients’ length of stay and adverse outcomes, improved patient progression and quality of care. However, they were uncertain if these benefits outweighed the cost of the weekend penalty rates staff needed to be paid.

Several allied health managers expressed firm beliefs that weekend allied health services were beneficial for seeing patients sooner and reducing patient length of stay, especially for those patients who could be discharged on the weekend or early in the following week. Allied health managers saw this as creating benefits for both the patient and the health service.

Manager 1: “There is a good body of research that shows that delays to care lead to less desirable outcomes. When people are stuck in the Emergency Department for 24 h there are less desirable outcomes, so part of it is making sure that you have beds available for people who need them, to get the care in a timely fashion—in the ward they should be getting it in.”

Another participant expressed doubt about this and described his frustration in not having clear evidence to base his decision on:

Manager 2: “I want to be guided by the research!!”

Not having a weekend service in some disciplines was thought to delay discharge. Managers described a “holding pattern” treatment regimen where a plan is set in place on a Friday to allow the patient to be safely managed by nursing staff over the weekend.

Manager 3: “With a weekend service, speech (pathologists) feel that they can upgrade someone on a Friday because they know they will be checked the next day…..and tend to be very conservative on a Friday if they do not have a weekend service. I have worked at (a service without weekend speech pathology) and we were always very conservative on a Friday because if you know (they) are not going to be checked until the Monday, that could be 2 days that they are brewing a chest (infection).”

Manager 4: “Also the trache(ostomy) patients … the Intensive Care Unit is 24 h care and then it just stops for 2 days (when the patient is moved to a ward without weekend allied health), so the progression of decannulation is halted by the fact that there is no speech pathologist here (on weekends).”

Managers described feedback from nursing and medical staff who reported that their work could be impeded if allied health were not available.

Manager 5: “(Without a weekend allied health service) someone is sitting there nil by mouth (fasting) and medical and nursing don’t like it. It holds back their ability to move their work forward in terms of how they administer medications.”

Reducing the risk of adverse patient events, such as falls, pressure injuries and pulmonary deterioration requiring transfer to intensive care, was seen as a key benefit of having a weekend service. Adverse events, defined as “unintended injuries caused by medical management rather than the disease process,” [27] can lead to long term disability or death, and also increase the length of stay and the cost of treatment [28].

Manager 6: “The weekend service can prevent post-operative pulmonary complications, improve respiratory function for patients who already have compromised respiratory function, [and] stop deterioration on the wards.”

Manager 7: discussing a period without weekend allied health: “The patient who had a massive tracheostomy, mandibulectomy, hemiglossectomy, fibula flap and skin graft and he didn’t mobilize for 4 days post operatively—the fact that he didn’t mobilize or sit out of bed, that could be a risk factor. I’ve seen these guys deteriorate quickly.”

Another potential benefit expressed by participants was an improved patient experience. This is demonstrated in a discussion about funding for Public Holiday services:

Manager 5: “… in X’s time she made a change, for budgetary reasons, to cease funding Public Holidays. So we have been putting (a service) in—in rather a reactionary fashion—when medical and nursing have requested it. Easter for example; they requested very late in the piece—after we said we weren’t going to have service—they asked for a service….. That’s why our vision is to have 365 day a year service is because we …. think to actually have someone nil by mouth (fasting) is just a really awful experience for a patient and so feel that, even it is a shortened service and we are just targeting those who are nil by mouth and safety checks and the new assessments, I think that is a very patient centred approach.”

Participants reported that weekend allied health services might enable a more comprehensive model of care that creates benefits beyond the patient in the hospital bed. For example, having a skilled social worker available on the weekend was seen to reduce the risk of harm to vulnerable individuals dependent on the patient.

Manager 8: “…(A) social worker did her psychosocial assessment with the patient and identified very quickly that there were kids at home and was able to identify that those kids were probably home with an unsafe adult…. someone who was a substance user, kids who were under ten, a whole lot of risks around what might happen.”

Weekend penalty rates mean that the service costs more per hour than weekday services. The higher cost of wages on weekends compared to weekdays was seen by all allied health managers as a principal disadvantage of providing weekend allied health service. This made some participants uncertain whether the benefits they thought were gained from having the weekend allied health service justified the costs.

Manager 2: “They are all getting casual pay—which is cheaper than employing full time (permanent) staff (to cover weekend shifts). You have to pay (permanent workers) double (on weekends) and…. if they do more than ten weekend shifts you’ve got to give them an extra week of annual leave so we don’t really do that.”

### Key uncertainty 2) Are we structuring our services the optimum way?

Several areas of uncertainty were raised relating to how services were structured and operated. Managers wondered if staff were seeing the right patients, if the discipline mix was right, if it was possible to predict when the service was most needed and whether the staff employed had the right experience to best provide a weekend service.

Some participants were doubtful whether some of patients they were currently seeing on their weekend list were actually experiencing the intended benefits.

Manager 9: “I think when the services are always running, people put (patients) on the (weekend) list. People would say *we’re going to see them Saturday and Sunday and the plan is they go home Monday or Tuesday* and I would say 95% of the time the patient is going home on Monday or Tuesday whether they meet their physiotherapy goals or not. I think it’s a real problem.”

Manager 2: “I’m really struggling to think of advantages compared to all the disadvantages! I think the wards get used to it (having a service). I’m not convinced that it’s worth the money and the time that goes into organizing it for certain groups.”

Managers also noted that inappropriate referral could lead to inefficient service delivery.

Manager 9: “[Nursing staff say] *we've got a physio here and we'll just get them to see them*. They are a percentage of the new referrals and they are the inappropriate referrals.”

Another questioned whether a weekend service was required on every weekend to the same degree.

Manager 9: “Do you staff a 24 h, 7 day physiotherapy service on the basis that you'll actually need the (extra) service one of two times every 3 months? Is it really worth it?”

One manager questioned the current mix of disciplines, given his hospital provided only occupational therapy and physiotherapy on weekends. He also recognised that he could provide more hours of cover if he chose to use the money during the week. When asked how he would improve the weekend allied health service in the second half of the larger trial, when a new service was to be developed for the research wards, he was happy to reduce his service and when asked what he would do with the money saved said:

Manager 2: “…Give it to other disciplines or use it during the week. We can get almost double….the equivalent of 4 days of a Grade 1 clinician for what we spend (on the acute medical and surgical wards) on the weekend.”

Two managers raised the idea of proving services by having staff on-call for telephone advice rather than paying staff to come to work on weekends.

Manager 10: “I think budget wise—I think the money could be spent better. I’m not sure if we need Saturday and Sunday. I think maybe it’s good to have someone here 1 day and then on call on the Sunday. Then they would know the complex patients—if there is going to be an issue with them—they are the ones I would put on a Sunday review as well. Then the new Sunday patients could just wait until Monday.”

Some questioned if the service was needed by all the patients who had previously been referred for weekend therapy. Physiotherapy managers described situations when their weekend staff were asked to perform tasks that could be performed by nursing staff, such as getting patients out of bed or checking that patients were safe to walk when this had previously been documented in the medical record. Other managers questioned the need for the service in their discipline.

Manager 11: “Sometimes I wonder if we really need to be here…… some things could be put in place on a Friday. Part of me thinks if we are so busy on a Friday, why not put someone on then, instead of on the weekend? Or if processes could be put in place instead of having staff come in to work?”

Participants also questioned the mix between junior and senior staff employed. Managers must choose between employing cheaper, less experienced staff and employing more experienced staff, possibly for fewer hours. The hidden cost of employing less experienced staff is that of training, with weekday staff spending many hours preparing junior staff for working weekends. Some managers felt that employing more experienced staff would improve the quality of care and potentially save money in training time.

Manager 9: “(Senior staff) are more willing to make different decisions and have a higher tolerance that the patient might not be functionally perfect before they leave the health service. It can be difficult for junior staff to shift the paradigm; if (patients) have been referred … for respiratory treatment….(the weekend therapists) don’t make the decision to assess or progress their mobility. You’d come back and they had had the same thing.”

Manager 12: “That’s when the junior staff can’t triage or even have the guts to go up to the doctor and say: “*how long are they going to be here?  Do we really need to see them today?*”

### Challenges experienced when providing a weekend allied health service

Participants described several challenges for staffing a weekend service. Chronologically, these challenges involve the recruitment, training, rostering, performance management, and retention of staff that work outside of normal business hours.

Manager 2: “So the time commitment in advertising and recruiting a work pool who are external—the rostering hours—it is much larger than sometimes I think the buy-back is.”

The cost and logistics of training weekend staff were mentioned by most managers as a challenge; none more so than the managers who only used junior staff on the weekend.

Manager 7: “The burden of training… one staff member to work on the weekend—I think it would be about four full days.”

Manager 13: “For the number of shifts they would do versus the turnover of juniors that we are constantly training, I suspect that it would potentially cheaper (to employ more experienced staff)!”

All managers employed staff from outside their health service on weekends to increase the staffing pool. Many spoke of the challenges resulting from managing staff who worked at a different time to themselves and the senior clinical team. All had processes in place to perform a rudimentary annual performance review by phone, but performance issues and gaps in clinical knowledge were harder to address.

Manager 11: “The organisation is not really set up for 7 days (of) service…. the staffing and management of the weekend staff takes more time….getting them to do competencies, performance enhancement, corporate induction—there are those logistical difficulties, workforce management stuff, that makes it more difficult.”

Manager 1: “Part of my issue is that if they are staff who don’t work with us, (if) we are not having those ongoing discussions, (or) formats for talking about patient care, then how are we going to know (what they are doing)?”

The employment of staff from outside the organisation was seen to have the advantage of filling shifts thus avoiding the extra penalty rates and leave requirement associated with rostering internal staff. Some managers, however, questioned the commitment of some external staff to their organisation.

Manager 2: “On the whole, weekend staff are poorly engaged with the organisation and the department—they just come in, do their job and go home. Even though they are getting Grade Two (pay) rates and all the extra loading.”

Another issue potentially reducing the efficiency of the weekend allied health service was that many services that allied health staff refer patients to for follow up care are not open on the weekend, both within the hospital [1, 7, 13] and in the community [29].

Manager 14: “Most of our services that we refer to are closed. So referrals are partially completed but then it can’t be faxed until Monday. So then we are handing back to weekday staff.…….In a situation where there was no family to provide meals, then she wouldn’t have been able to go home and a social worker wouldn’t have been able to do anything about it. We can’t access *Meals-on-Wheels* until Monday.”

## Discussion

This is the first study to examine the experiences of allied health managers in the managing of and training for the staff that deliver weekend allied health services. It has revealed two major uncertainties faced by managers when providing weekend allied health services on acute medical and surgical wards: “Do the costs outweigh the benefits?” and “What type of service should be provided?” These uncertainties indicate there may be great variation in how these services are delivered in real life and in the actual cost-effectiveness of these real life programs. The challenges described by the participants in this study imply that running a sustainable and efficient weekend service is very difficult, given the problems with recruiting, training, rostering and retaining staff and the limitations to other service availability on the weekends.

A number of managers perceived benefits to both patients and the health service in providing weekend allied health services and saw the decision to provide these services, despite the challenges, as important part of their commitment to patient care. Managers perceived that by providing specialist services earlier, they may be potentially reducing patient length of stay. The quality and safety of patient care was perceived to be enhanced by providing services such as swallowing assessments over weekends, potentially allowing some patients to start food and fluid after a period of fasting. Evidence that weekend allied health services result in these improved patient outcomes is yet to be produced.

The challenges that manager faced in the recruitment, training, rostering, performance management, and retention of staff that work outside of normal business hours, together with the costs, resulted in some of the managers questioning if the service was worth providing. These difficulties were despite the services being provided for significantly fewer hours than those provided during the week. The challenges in providing weekend services have also been experienced in England, where exploration of a full 7 day per week medical services has been advocated [30]. The cost of providing the same levels of medical staffing as during the week has been estimated as £900 m per year after the “benefits such as reduced length of stay and reduced admissions” are taken into account [31]. Plans to move junior doctors onto contracts to deliver more weekend services were strongly opposed by medical groups in England [32].

Some services that are currently provided by allied health on the weekend could potentially be provided by other professions. Dysphagia screening after acute stroke completed by trained nurses has been found to reduce wait times for screening from 35 h to less than 1 h [33]. Malnutrition can be recognised by medical and nursing staff using validated nutrition screening tools [34]. Nurse led early mobilisation and enhanced oral intake after colonic resection has been shown to reduce the length of hospital stay in high risk patients [35]. These studies have not involved directly comparing the outcomes services provided by allied health and nursing professionals or focussed on the provision of services on weekends.

This research highlights a perceived need of some allied health managers for research examining the cost-effectiveness of weekend allied health services and investigating how they should be structured. An economic modelling study from the UK reported that providing a 7 day medical services in emergency and admission units would add 1.5 to 2% to the cost of care, but this would cost only £20,000 per quality adjusted life year gained [36]. An Australian randomised trial found that adding a Saturday physiotherapy and occupational therapy service in the rehabilitation setting resulted in a cost saving per quality adjusted life year gained and a clinically important gain in functional independence [37]. There have, however, been no randomised trials demonstrating that weekend allied health services are effective or cost-effective when provided in the acute setting. The literature around the structure of weekend allied health services is also limited. Cross-sectional research from Australian [15] and Canadian hospitals [16] indicate how many hospitals have services on weekend days, but do not advise how they should be structured to optimise outcomes.

This study contained several strengths and limitations that should be considered. We examined the provision of weekend allied health from the perspective of the service managers. Managers from a variety of disciplines were interviewed from two large metropolitan health services with existing weekend services to acute medical and surgical wards. The views of the multidisciplinary ward teams were not included in this paper but will be reported separately. The views of patients and their families were not explored. The data were gathered and analysed by a team which consisted mainly of allied health professionals, some of whom work at the hospitals studied. This may have affected the collection and interpretation of data. To counteract this, and to improve trustworthiness, a researcher external to both the health services and allied health was included in the research team to assist with development of the data collection approach and interpretation. Personal biases were discussed between the principal investigator and the external researcher and strategies to limit their potential impact developed and implemented.

Well-designed randomised controlled trials are needed to fully explore the effectiveness and cost effectiveness of providing weekend allied health services whilst also considering the cost of providing these services. Measures could include patient length of stay, rates of adverse outcomes such as falls and pressure injuries, and unplanned patient readmissions within a set period after discharge. A dose response study of weekend allied health services would provide health services managers with data to help understand the optimal resources that should be allocated to provision of a weekend service on acute wards. Even then, such a study would be limited by numerous possible variations in how that amount of resource could be divided between the professional disciplines and which patients could be targeted as a priority. Pragmatic studies that examine the effectiveness and cost-effectiveness of service models as they are provided in real life contexts would also provide vital insights as to whether decision makers will receive good value for money by investing in these services. Studies into the effect of the experience levels of allied health staff on patient outcomes and other staffing models would also be beneficial. This information would assist to reduce the uncertainty faced by allied health managers by informing their decisions in planning weekend allied health services and may reduce the variation between services provided.

## Conclusion

Weekend allied health services are seen by their managers as adding value to the health service by providing timely service to those patients needing specialist allied health services on the weekend, reducing patient length of stay, adverse incidents, and improving the quality of care. The managers also recognise the high cost of providing these services is relatively high, given the penalty wages, the hidden costs of managing the service, and the inherent inefficiencies involved in providing out of hours care. In an environment of rising healthcare costs such as Australia, there is opportunity for public and professional debate about the most effective way of providing weekend allied health care services, particularly in health services who provide limited other weekend services. Despite demand for 7 day a week service from consumers and manager perceptions of improved patient quality of care, without studies which show weekend allied health services on acute medical and surgical wards clearly change patient outcomes or provide health economic gains, these resources may need to be redirected. The resources may be better spent in areas with clear evidence to show the addition of weekend allied health services improves patient outcomes, such as on acute assess units and rehabilitation wards.

## Appendix

**Table 3:** Interview questions

Questions	
1	What do you think are the advantages of having a weekend allied health service?
	What sorts of patients most benefit from your current weekend allied health service and how?
2	What are the disadvantages of the weekend allied health service?
	What sorts of patients benefit the least from your current weekend allied health service and why?
3	Are there any other issues?
	Staffing, supervision, training, handovers to and from weekday staff
4	How have weekend allied health services developed for your discipline?
	• Evidence?
	• Experience?
5	What concerns do you have about the withdrawal of the weekend allied health service?
	What is the reasoning behind these concerns? Are they based on evidence, experience or something else?

## References

[1] Barba R, Losa JE, Velasco M, Guijarro C, García de Casasola G, Zapatero A (2006). Mortality among adult patients admitted to the hospital on weekends. Eur J Intern Med.

[2] Daly T. Evenings, nights and weekends: working unsocial hours and penalty rates. In: Centre for work and life. Adelaide: University of South Australia; 2014.

[3] Australian Nursing and Midwifery Federation. Hands off our penalty rates. Lamp. 2015;722:28–9

[4] Hamermesh D. Do labor costs affect companies’ demand for labor? IZA World Labor. 2014;4:1–10.

[5] Lee LH, Swensen SJ, Gorman CA, Moore RR, Wood DL (2005). Optimizing weekend availability for sophisticated tests and procedures in a large hospital. Am J Managed Care.

[6] Redelmeier DA, Bell CM (2007). Weekend worriers. New Engl J Med.

[7] Schmulewitz L, Proudfoot A, Bell D (2005). The impact of weekends on outcome for emergency patients. Clin Med J R Coll Phys Lond.

[8] Albright KC, Raman R, Ernstrom K, Hallevi H, Martin-Schild S, Meyer BC, Meyer DM, Morales MM, Grotta JC, Lyden PD (2009). Can comprehensive stroke centers erase the ‘weekend effect’?. Cerebrovasc Dis.

[9] Buckley D, Bulger D (2012). Trends and weekly and seasonal cycles in the rate of errors in the clinical management of hospitalized patients. Chronobiol Int.

[10] Cram P, Hillis SL, Barnett M, Rosenthal GE (2004). Effects of weekend admission and hospital teaching status on in-hospital mortality. Am J Med.

[11] Bell CM, Redelmeier DA (2004). Waiting for urgent procedures on the weekend among emergently hospitalized patients. Am J Med.

[12] Maggs F, Mallet M (2010). Mortality in out-of-hours emergency medical admissions--more than just a weekend effect. J R Coll Physicians Edinb.

[13] Lee KG, Vaithilingam I (2012). A study of weekend and off-hour effect on mortality in a public hospital in Malaysia. Med J Malays.

[14] Mohammed MA, Sidhu KS, Rudge G, Stevens AJ (2012). Weekend admission to hospital has a higher risk of death in the elective setting than in the emergency setting: a retrospective database study of national health service hospitals in England. BMC Health Serv Res.

[15] Shaw KD, Taylor NF, Brusco NK (2013). Physiotherapy services provided outside of business hours in Australian hospitals: a national survey. Physiother Res Int.

[16] Campbell L, Bunston R, Colangelo S, Kim D, Nargi J, Hill K, Brooks D (2010). The provision of weekend physiotherapy services in tertiary-care hospitals in Canada. Physiother Can.

[17] Brusco NK, Shields N, Taylor NF, Paratz J (2007). A Saturday physiotherapy service may decrease length of stay in patients undergoing rehabilitation in hospital: a randomised controlled trial. Aust J Physiother.

[18] Davidson I, Hillier VF, Waters K, Walton T, Booth J (2005). A study to assess the effect of nursing interventions at the weekend for people with stroke. Clin Rehabil.

[19] DiSotto-Monastero M, Chen X, Fisch S, Donaghy S, Gomez M (2012). The efficacy of seven days per week inpatient admissions and rehabilitation therapy. Arch Phys Med Rehabil.

[20] Peiris CL, Shields N, Brusco NK, Watts JJ, Taylor NF (2013). Additional Saturday rehabilitation improves functional independence and quality of life and reduces length of stay: a randomised controlled trial. BMC Med.

[21] Hakkennes S, Lindner C, Reid J (2015). Implementing an inpatient rehabilitation Saturday service is associated with improved patient outcomes and facilitates patient flow across the health care continuum. Disabil Rehabil.

[22] Neergaard MA, Olesen F, Andersen RS, Sondergaard J (2009). Qualitative description–the poor cousin of health research?. BMC Med Res Methodol.

[23] Haines T, O’Brien L, Mitchell D, Bowles K-A, Haas R, Markham D, Plumb S, Chiu T, May K, Philip K (2015). Study protocol for two randomized controlled trials examining the effectiveness and safety of current weekend allied health services and a new stakeholder-driven model for acute medical/surgical patients versus no weekend allied health services. Trials.

[24] Braun V, Clarke V (2006). Using thematic analysis in psychology. Qualitative Res Psychol.

[25] Braun V, Clarke V: What can “thematic analysis” offer health and wellbeing researchers? Int J Qualitative Stud Health Well-Being. 2014;9:1–2. 10.3402/qhw.v9.26152 PMC4201665 25326092

[26] Firth J, Smith J (2011). Qualitative data analysis: the framework approach. Nurse Res.

[27] Vincent C, Neale G, Woloshynowych M (2001). Adverse events in British hospitals: preliminary retrospective record review. BMJ.

[28] Ehsani JJ, Terri, Duckett S (2006). The incidence and cost of adverse events in Victorian hospitals 2003–04. Med J Aust.

[29] Boxall A-m, Sayers A, Caplan GA (2004). A cohort study of 7 day a week physiotherapy on an acute orthopaedic ward. J Orthopaedic Nurs.

[30] Freemantle N, Ray D, McNulty D, Rosser D, Bennett S, Keogh BE, Pagano D (2015). Increased mortality associated with weekend hospital admission: a case for expanded seven day services?. BMJ.

[31] Torjesen I (2016). Seven day services will need 4000 extra doctors, says leaked report. BMJ.

[32] Moberly T (2016). Junior doctors’ strikes: BMA responds to your questions. Br Med J Publishing Group.

[33] Lees L, Sharpe L, Edwards A (2006). Nurse-led dysphagia screening in acute stroke patients. Nurs Stand.

[34] Kruizenga HM, Van Tulder MW, Seidell JC, Thijs A, Ader HJ (2005). Effectiveness and cost-effectiveness of early screening and treatment of malnourished patients. Am J Clin Nutr.

[35] Basse L, Jakobsen DH, Billesbolle P, Werner M, Kehlet H (2000). A clinical pathway to accelerate recovery after colonic resection. Ann Surg.

[36] Crump H (2015). Seven day working: why the health secretary’s proposal is not as simple as it sounds. BMJ.

[37] Brusco NK, Watts JJ, Shields N, Taylor NF (2014). Are weekend inpatient rehabilitation services value for money? An economic evaluation alongside a randomized controlled trial with a 30 day follow up. BMC Med.

